# COVID-19 pandemic and gynaecological endoscopic surgery

**Published:** 2020-05-07

**Authors:** Ertan Saridogan, Grigoris Grimbizis

On the last day of 2019, the World Health Organization (WHO) China Country Office was notified of cases of pneumonia of unknown aetiology detected in Wuhan City, Hubei Province of China. The Chinese authorities quickly identified the cause as a new type of coronavirus on 7 January 2020 and the infection was named as Coronavirus Disease-2019 or COVID-19. The virus spread around the world rapidly and WHO declared it as a pandemic on 11 March 2020 (World Health Organization, 2020). This global pandemic has changed the way we live our lives and had a major impact on the healthcare services and professionals. Some of these changes may be long lasting and may change the way we practice our profession in the long term. Currently governments, nations, international and national organisations, industry and health services are trying to reduce the speed of viral spread and trying to limit its morbidity and mortality. Many countries are in either complete or partial lockdown. Regardless of the structure they previously had, healthcare services have switched to a centralised, government led organisation. They have diverted their resources to looking after patients infected by the coronavirus. Even private healthcare facilities have been made available to state healthcare systems to aid the fight with this pandemic in some countries.

Healthcare services have cancelled elective operations and minimised hospital attendances for face-to-face consultations. Laparoscopic surgery was quickly flagged up as a potential area where the risk of transmission might be higher in patients with the COVID-19 infection (Royal College of Surgeons, 2020). This has naturally caused some disquiet amongst surgeons. National and international bodies have published recommendations to advise how to organise services and what precautions to take for gynaecological surgery to limit transmission and protect healthcare professionals, whilst providing the essential care to patients (British Society for Gynaecological Endoscopy, 2020, European Society for Gynaecological Endoscopy, 2020, Royal College of Obstetricians and Gynaecologists, British Society for Gynaecological Endoscopy and British Gynaecological Cancer Society, 2020). All of these recommendations recognised the scarcity of evidence or data specific to coronavirus infection in relation to abdominal surgery. In this issue of *Facts, Views and Vision*, the article by Mallick et al. (2020) summarises what is known in relation to gynaecological laparoscopic surgery and highlights the unknowns. The authors emphasise that there is a theoretical but unproven risk of transmission during laparoscopic procedures because the viral RNA is present in the blood of 1-15% of the patients and that presence of artificial pneumoperitoneum is likely to generate aerosol due to escape of CO 2 which may contain the virus within droplets of blood or the surgical smoke. A more recent review of COVID-19 patients showed that, in fact, the viral RNA in blood is found in almost all (96.8%) of patients included in the publications before 23 February 2020 (Rodrigues-Morales et al., 2020).

The virus which is causing the current pandemic is Severe Acute Respiratory Syndrome Coronavirus-2 (SARS-CoV-2) which is a member of the β coronaviruses (CoVs). CoVs are RNA viruses and commonly cause upper respiratory infections in humans. Novel coronaviruses SARS-CoV and MERS-CoV, which emerged in 2002 and 2012 respectively, caused severe lower respiratory tract infections. Viral RNA of both these types were found in the plasma during the acute phase, but the live MERS-CoV was not isolated (Chang et al., 2020). Hence, it is unclear if the viral particles in the blood have the capacity to infect other people.

SARS-CoV-2 RNA was detected in the blood of most cases but the viral RNA load was found to be very low (Chang et al., 2020). This raises further questions as to whether there is a real risk of transmission of infection from exposure to blood either in the form of air droplets or surgical smoke during surgery. Mallick et al. (2020) extrapolate that there may be possible transmission due to exposure to surgical smoke from Hepatitis B (HBV), Human Immunodeficiency Virus (HIV) and Human Papilloma Virus (HPV). They do, however, admit that this risk remains mostly theoretical and controversial. There are no documented cases of HBV or HIV transmission from the surgical smoke. There are four cases of HPV transmission; HPV positive laryngeal papillomatosis or oropharyngeal squamous cancer were reported in healthcare professionals who had no risk factors other than repetitive exposure to surgical smoke in the literature (Liu et al., 2019). Whilst the overall risk remains low, the possibility of transmission from surgical smoke may be related to the specific transmission route of the virus in general; blood borne viruses may not be able to infect but an orogenital virus such as HPV can. If this is true, then there is a chance that SARS-CoV-2 may have the potential to infect the respiratory tract from the surgical smoke, if full live viral particles are present in it.

Another important aspect of laparoscopic surgery is the escape of surgical smoke to the theatre environment. There has been a lot of debate over this point and this is used by some to justify open surgery over laparoscopy. Surgical smoke is produced during both open and laparoscopic surgery. In fact, laparoscopy may offer an advantage over open surgery on this issue; the smoke is collected in a confined space, and as long as the smoke is evacuated safely, escape to the theatre environment may be much less compared to open operations. During open procedures, smoke inevitably dissipates into the theatre environment in an uncontrolled manner, even when effective suction devices are used.

Whilst there is uncertainty about the transmission through surgical smoke or the escaping CO_2_ during laparoscopic surgery, what is clearer is that the virus is more likely to infect healthcare professionals during intubation or extubation for general anaesthesia (anaesthetic team), or during procedures involving the upper respiratory tract (such as Ear-Nose-Throat surgeons). Hence, general anaesthesia appears to be the dominant risk factor when a gynaecological operation is needed and the recommendations from various organisations recognised the need for personal protective equipment for theatre personnel. Avoiding general anaesthesia when possible is probably a sensible step in reducing the risk of transmission. Whilst this might be impossible for laparoscopic surgery, certain emergency gynaecological procedures such as ruptured ectopic pregnancy or ovarian torsion can probably be performed via minilaparotomy under regional anaesthesia, in the absence of other risk factors such as obesity. Most hysteroscopic procedures can also be performed without general anaesthesia, either as office procedures or under sedation without intubation, minimising the hazard to the operating team.

The ESGE recommendations on Endoscopic Surgery (European Society for Gynaecological Endoscopy, 2020), also published in this issue, highlight the importance of screening for SARS-CoV-2 before gynaecological procedures, when possible. There may not be enough time to screen women for the virus in emergency situations, but when there is time this seems to be a very logical approach. However, we need to recognise the limitations of currently available tests. Although the reverse transcriptase – polymerase chain reaction (RT-PCR) tests appear to be 100% specific, false negative rates of 47-70% have been reported from oropharyngeal and nasopharyngeal swabs (Alhazzani et al., 2020). Hence a single negative test does not rule out the infection. Some hospitals combined RT-PCR testing with further imaging (chest X-ray or CT) to enhance the detection rates, but imaging is less likely to be useful in asymptomatic patients, or those with mild symptoms. It is likely that the sensitivity of RT-PCR tests will gradually improve and continuing to use it in combination with screening for symptoms and imaging looks like a sensible approach.

In conclusion, we are left with many unknowns as regards to the risk of COVID-19 transmission during gynaecological endoscopic surgery. The initial COVID-19 specific publications have mostly originated from China, but more reports are now being disseminated from the rest of the world. Thus, as more data accrue and our knowledge of the impact and behaviour of this novel virus becomes greater, recommendations may need to be revised. However, we probably will not have the answers to most of the questions that have been raised about gynaecological endoscopic surgery during the course of the pandemic and it is quite likely that our understanding will be enhanced after the outbreak is over. Meanwhile, it looks sensible to take reasonable precautions, including theuse of appropriate personal protective equipment and taking precautions to reduce exposure to escaping CO_2_ or surgical smoke during the pandemic.

Ertan Saridogan, Editor, Facts, Views and Vision, University College London HospitalsGrigoris Grimbizis, President, ESGE, Aristotle University of Thessaloniki
